# Supplementary material for: Use of volatile anesthetics for sedation in the ICU during the COVID-19 pandemic: A national survey in France (VOL’ICU 2 study)
Source: PLoS One. 2022 Dec 29;17(12):e0278090. doi: 10.1371/journal.pone.0278090 (PMC9799316; doi:10.1371/journal.pone.0278090)

**Use of Volatile anesthetics for sedation in the ICU during the COVID-19 pandemic: A national survey in France (**VOL’ICU 2 study**)**

Raiko Blondonnet^1,2^, Aissatou Balde^1^, Ruoyang Zhai^2^, Bruno Pereira^3^, Emmanuel Futier^1,2^, Jean-Etienne Bazin^1^, Thomas Godet^1^, Jean-Michel Constantin^4^, Céline Lambert^3^, Matthieu Jabaudon^1,2,5^

##

**Corresponding author**

Raiko Blondonnet, MD , MSc

## Department of Perioperative Medicine, CHU Clermont-Ferrand; GReD, CNRS, INSERM, Université Clermont Auvergne; 1 Place Lucie Aubrac, 63003 Clermont-Ferrand Cedex 1, France. (Mail) [rblondonnet@chu-clerm ontferrand.fr](mailto:rblondonnet@chu-clermontferrand.fr)

## **Additional files**

**Additional file: Supplemental Content 1.** Survey questionnaire

1. **General characteristics of the ICU**

Your place of professional exercise:

- University Hospital
- General Hospital
- Private health structure
- Other

In which type of intensive care unit do you work?

- Medico-surgical / Polyvalent
- Medical
- Step-down unit
- Medical intensive care, such as a coronary care unit
- Other

In which French region do you work?

- Auvergne - Rhône-Alpes
- Bourgogne-Franche-Comté
- Bretagne
- Centre-Val de Loire
- Corse
- Grand-Est
- Hauts-de-France
- Ile-de-France
- Normandie
- Nouvelle-Aquitaine
- Occitanie
- Pays de la Loire
- Provence-Alpes-Côte d’Azur
- Corse
- DOM-TOM

Number of senior physicians in your unit (full-time equivalent): (numerical value)

Number of residents in your unit: (numerical value)

Number of beds in your intensive care unit: (numerical value)

Number of step-down beds in your unit: (numerical value)

Number of patients admitted per year in your intensive care unit: (numerical value)

Have you admitted COVID-19 patients since january 2020 ?

- No
- Yes:

What percentage of patients are admitted with ARDS in your unit?

- during pandemic peak of spring 2020 (numerical value)
- during pandemic peak of autumn 2020 (numerical value)
- during pandemic peak of winter 2021(numerical value)
- between the pandemic peaks(numerical value)

What percentage of patients are admitted with COVID-19 in your unit since january 2020?

- during pandemic peak of spring 2020 (numerical value)
- during pandemic peak of autumn 2020 (numerical value)
- during pandemic peak of winter 2021(numerical value)
- between the pandemic peaks(numerical value)

What is the average length of stay in the intensive care unit (in days) of your Non-COVID-19 patients? (numerical value)

What is the average length of stay in the intensive care unit (in days) of your COVID-19 patients? (numerical value)

What is the average duration (in days) of invasive mechanical ventilation in your Non-COVID-19 patients? (numerical value)

What is the average duration (in days) of invasive mechanical ventilation in your COVID-19 patients? (numerical value)

What is the mortality rate, in the intensive care unit, of your Non-COVID-19 patients? (numerical percentage value)

What is the mortality rate, in the intensive care unit, of your COVID-19 patients? (numerical percentage value)

1. **General data on inhaled sedation use**

Are you familiar with inhaled ICU sedation?

- No
- Yes, before the COVID_19 pandemic:
  - I am familiar with the AnaConDa System
  - I am familiar with the Mirus System
  - I am familiar with another system
- Yes, since the COVID_19 pandemic:
  - I am familiar with the AnaConDa System
  - I am familiar with the Mirus System
  - I am familiar with another system

Do you have dedicated systems to deliver inhaled sedation available in your institution?

- No
- Yes, before the COVID_19 pandemic:
  - I am familiar with the AnaConDa System
  - I am familiar with the Mirus System
  - I am familiar with ventilator from the operating room to deliver halogenated agents
  - I am familiar with another system
- Yes, since the COVID_19 pandemic:
  - I am familiar with the AnaConDa System
  - I am familiar with the Mirus System
  - I am familiar with ventilator from the operating room to deliver halogenated agents
  - I am familiar with another system

Did you use inhaled sedation in your ICU before the COVID-19 pandemic?

- Never
- Sometimes
- Often

Do you use inhaled sedation in your ICU since the COVID-19 pandemic?

- Never
- Sometimes
- Often

Has your use of inhaled sedation changed since the COVID-19 pandemic?

- No
- Yes
  - I use inhaled sedation more frequently in all patients
  - I use inhaled sedation more frequently in ARDS patients
  - I use inhaled sedation because of the shortage of intravenous sedative
  - I stop to use inhaled sedation
  - Other

If you don’t use inhaled sedation, do you think you will develop inhaled sedation in your unit in the next two years ?

What are the reasons to use inhaled sedation since the beginning of COVID-19 pandemic ?

- Shortage of intravenous sedatives
- Add sedative
- Involvement in a clinical trial about inhaled sedation
- Curiosity
- Other

Who uses inhaled sedation in your ICU?

- Nobody
- Some physicians
- All physicians

In how many patients per year do you use inhaled ICU sedation before the COVID-19 pandemic?

- <20
- 20-50
- 50-100
- >100

In how many patients per year do you use inhaled ICU sedation since the COVID-19 pandemic?

- <20
- 20-50
- 50-100
- >100

If you do not use inhaled ICU sedation, what are the reasons? (multiple answers possible)

- No equipment available
- Untrained medical staff
- Untrained paramedical personnel
- No obvious interest
- Heavy organization
- Concerns about air pollution risk or ecological impact
- Undesirable effects
- Lack of habit
- Cost issues

If you use inhaled sedation in your ICU, what is/are the indication(s) for use? (multiple answers possible

- COVID-19 patient only
- Medical
- Post-surgery
- ARDS
- Traumatology
- Failure of IV sedation (drug or alcohol addicts, etc.)
- Asthma
- Cardioprotection
- Status epilepticus
- Neuroprotection
- No specific indication
- Other(s)

In your opinion, what are the potential benefits of inhaled ICU sedation? (multiple answers possible)

- I don't know
- Ease of use, including “on/off” effects
- Bronchodilating properties
- Anti-inflammatory properties
- Low cost
- Other(s)

1. **Practical aspects of inhaled ICU sedation**

Do you have a written protocol for inhaled sedation in your ICU?

- No
- Yes

Do you have any specific training in inhaled ICU sedation?

- No
- Yes

What halogenated agent do you use? (multiple answers possible)

- Sevoflurane
- Isoflurane
- Desflurane

For what reason(s) do you use this (these) halogenated agent(s)? (multiple answers possible)

- Availability
- Cost
- Ease of use
- Low metabolism
- Other(s)

With which mode(s) of ventilation do you use inhaled sedation? (multiple answers possible)

- Volume-controlled or pressure-controlled ventilation
- Pressure support (invasive) ventilation
- Pressure support (noninvasive) ventilation
- Other(s)

Do you usually combine an opioid agent with inhaled sedation?

- No
- Yes

If yes, which one(s)? (multiple answers possible)

- Sufentanil
- Fentanyl
- Remifentanil
- Other(s)

Do you usually combine another sedative agent when you use inhaled ICU sedation?

- No
- Yes

If yes, which one(s)? (multiple answers possible)

- Midazolam
- Propofol
- Ketamine
- Dexmedetomidine
- Other(s)

With what exhaled fraction of halogenated agents do you usually initiate inhaled ICU sedation?

- For sevoflurane: (numerical value)
- For isoflurane: (numerical value)
- For desflurane: (numerical value)

What expired fraction of halogenated agents do you typically target for "deep" ICI sedation?

- For sevoflurane: (numerical value)
- For isoflurane: (numerical value)
- For desflurane: (numerical value)
- Non applicable
- No expired fraction is targeted, I adapt sedation based on a score
- I only set a maximal expired fraction for safety reasons

How do you usually monitor the depth of sedation? (multiple answers possible)

- Sedation scale such as the RASS
- Monitoring of expired gas fraction
- Use of the Bispectral index (BIS)
- Other(s) :

Do you usually perform plasma assays for halogenated agents and/or their derivatives?

- No
- Yes

When do you usually stop inhaled sedation?

- When weaning off the ventilator
- During extubation
- Systematically at 48h
- No specific time limit
- Other(s)

In your opinion, what are the absolute contraindications of inhaled sedation?

- I don't know
- Acute renal failure
- Liver failure
- Pregnancy
- History of malignant hyperthermia
- Intracranial hypertension
- Other(s)

Have you ever had any serious adverse events that you believe were caused by inhaled sedation in ICU patients?

- No
- Yes

If yes, please detail: (multiple answers possible)

- Malignant hyperthermia
- Acute renal failure
- Severe respiratory acidosis
- Other(s)

What is your overall satisfaction with inhaled ICU sedation before the COVID-19 pandemic?

- Not satisfied at all
- Unsatisfied
- Indifferent
- Satisfied
- Very satisfied

What is your overall satisfaction with inhaled ICU sedation since the COVID-19 pandemic ?

- Not satisfied at all
- Unsatisfied
- Indifferent
- Satisfied
- Very satisfied

In your opinion, is inhaled sedation an interesting alternative to intravenous sedation in the intensive care setting?

- No
- Yes

In your opinion, is inhaled sedation an interesting alternative to intravenous sedation for COVID-19 patients in the intensive care setting?

- No
- Yes

Free comments, if any:

**Additional file: Supplemental Content 2.** Characteristics of the centers where respondents worked and characteristics of the non-COVID-19 and COVID-19 patients estimated by the respondents (n=102). *Data are expressed as median [1st quartile; 3rd quartile] and means (SD). Percentages were rounded to the nearest whole number depending on whether the value after the decimal was greater than or less than 5.*

| VARIABLE | VOL’ICU 2  (n=102) |
| --- | --- |
|  |  |
| **Number of physicians per ICU (n)** | |
| Senior physicians | 7 [5-9] |
| Residents | 5 [3-7] |
| **Number of beds per ICU** **(n)** | |
| ICU beds | 12 [8-16] |
| Step-down units beds | 6 [4-8] |
| **Patients per year (n)** | 739 [550-1000] |
| **Patients with ARDS (%)** |  |
| *during spring 2020* | 80 [40-100] |
| *during autumn 2020* | 70 [40-80] |
| *during winter 2021* | 60 [30-80] |
| *between epidemic peak* | 20 [10-65] |
| **Patient with COVID-19 (%)** |  |
| *general* | 30 [20-60] |
| *during spring 2020* | 78 [39-100] |
| *during autumn 2020* | 70 [36-85] |
| *during winter 2021* | 68 [33-90] |
| *between epidemic peaks* | 10 [1-20] |
| **Duration of ICU stay (days)** |  |
| Non-COVID-19 patients | 6 [5,3-8] |
| COVID-19 patients | 15 [12-20] |
| **Duration of mechanical ventilation (days)** |  |
| Non-COVID-19 patients | 5 [4-7] |
| COVID-19 patients | 14 [10-15] |
| **Mortality (%)** |  |
| Non-COVID-19 patients | 19 [16-23] |
| COVID-19 patients | 25 [19-32] |

**Additional file: Supplemental Content 3.** Geographical distribution and epidemiological data on respondents (n=102). *DOM-TOM: Département d’outre-mer-Territoire d’outre-mer.*


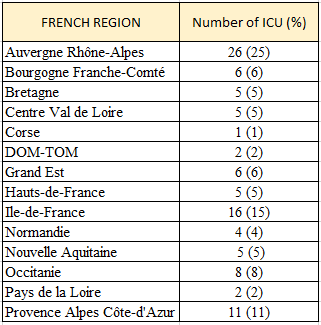

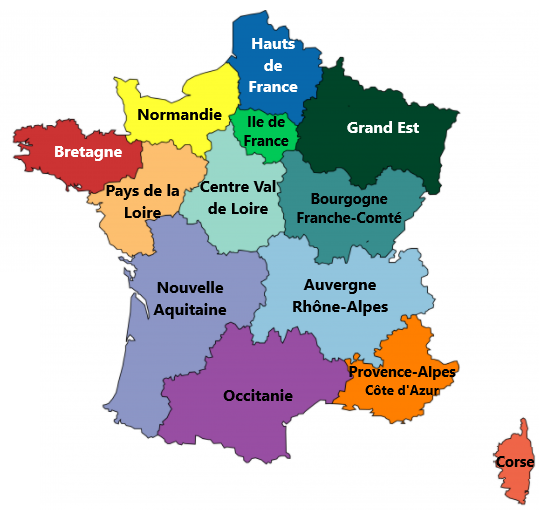


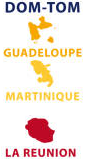

Supplement: S1 File — (DOCX) [file pone.0278090.s002.docx]
